# The Application of Combined Pre-Treatment with Utilization of Sonication and Reduced Pressure to Accelerate the Osmotic Dehydration Process and Modify the Selected Properties of Cranberries

**DOI:** 10.3390/foods8080283

**Published:** 2019-07-24

**Authors:** Malgorzata Nowacka, Artur Wiktor, Magdalena Dadan, Katarzyna Rybak, Aleksandra Anuszewska, Lukasz Materek, Dorota Witrowa-Rajchert

**Affiliations:** Department of Food Engineering and Process Management, Faculty of Food Sciences, Warsaw University of Life Sciences, Nowoursynowska 159c, 02-776 Warsaw, Poland

**Keywords:** cranberries, reduced pressure, sonication, color, bioactive compounds

## Abstract

The aim of this study was to investigate the effect of a pretreatment, performed by a combined method based on blanching, ultrasound, and vacuum application, on the kinetics of osmotic dehydration and selected quality properties such as water activity, color, and bioactive compound (polyphenols, flavonoids, and anthocyanins) content. The pretreatment was carried out using blanching, reduced pressure, and ultrasound (20 min, 21 kHz) in various combinations: Blanching at reduced pressure treatment conducted three times for 10 min in osmotic solution; blanching with reduced pressure for 10 min and sonicated for 20 min in osmotic solution; and blanching with 20 min of sonication and 10 min of reduced pressure. The osmotic dehydration was performed in different solutions (61.5% sucrose and 30% sucrose with the addition of 0.1% of steviol glycosides) to ensure the acceptable taste of the final product. The changes caused by the pretreatment affected the osmotic dehydration process by improving the efficiency of the process. The use of combined pretreatment led to an increase of dry matter from 9.3% to 28.4%, and soluble solids content from 21.2% to 41.5%, lightness around 17.3% to 56.9%, as well as to the reduction of bioactive compounds concentration until even 39.2% in comparison to the blanched sample not subjected to combined treatment. The osmotic dehydration caused further changes in all investigated properties.

## 1. Introduction

Cranberries are known as fruits rich in bioactive compounds, which provide positive effects and health benefits to the human body. The abundance of bioactive compounds contained in cranberries are also used in a medicine to treat urinary tract infections, gastrointestinal diseases, and support the neurological and cardiovascular conditions. Moreover, cranberries exhibit antivirus, anti-inflammation, and anticancer effects [1]. However, fresh cranberry is very sour, which is why its direct consumption and application is limited. Therefore, a sugar addition is required during processing in order to improve the taste of the fruit and to achieve consumers’ acceptance. On the one hand, osmotic dehydration (OD) is a process in which sugars are used to partially remove water from the material, and as an introduction of ingredients of the osmotic solution, usually sucrose, which has a beneficial effect on changing the flavor profile of the product. On the other hand, one of the main disadvantages of OD is time consumption [2].

Non-thermal methods such as pulsed electric fields, sonication, or high hydrostatic pressure have the potential to accelerate the OD kinetics. Among these methods, sonication is one of the technologies which can be used in OD intensification. It could be introduced as a pretreatment step or directly during the process [3,4,5,6]. Ultrasounds are series of vibrations at frequency varying between 20 kHz and 100 MHz, which spread in a given medium, i.e., in liquids, solids, or gases. The use of high power ultrasound leads to emergence of acoustic waves, which produce a direct (so-called “sponge effect”) and an indirect effect (cavitation) on solid food matrices [4,7,8]. Ultrasonic waves have an impact on nutritional and physico-chemical parameters of food, which are related to good extraction and the recovery of bioactive compounds [9,10,11,12,13]. It has been reported that high power ultrasound has been used to accelerate processes of mass exchange such as OD [5,14,15,16]. Moreover, ultrasound can be used alone or combined with different techniques [10].

Another promising method of OD facilitation is vacuum impregnation/infusion technology, which can also be described as a reduced pressure treatment. This method allows the air present in the plant pores to be replaced by acting solution in an instant. Due to the use of reduced pressure, mass transfer occurs faster, which is related to a hydrodynamic mechanism [17] and deformation-relaxation phenomena [18]. Isotonic or hypertonic solutions and other solutions containing valuable compounds beneficial for human health can be used for vacuum impregnation [19]. Similarly, OD might be performed under low-pressure conditions, which have a positive effect on shortening the process time in comparison to normal conditions. The use of low pressure during the treatment degasses the tissue, which increases the contact surface of the osmotic substance with the product, and the process of dehydration takes place faster [20].

In recent years, an increase in the number of obese people has been observed, therefore it is advisable to design production processes that enable reduction or replacement of sugar in food products [21]. Consumers poorly perceive the use of synthetic sweeteners, which is why food manufacturers are increasingly willing to use natural sweeteners, for example, steviol glycosides extracted from stevia leaves (*Stevia rebaudiana*). These leaves also contain nutritional and antioxidant compounds such as vitamin C, polyphenols, carotenoids, chlorophylls, and other macro and micronutrients [22,23,24]. Thanks to the application of steviol glycosides, it is possible to reduce sugar content while maintaining proper sweetness of the product [25,26,27]. However, it is usually used as an addition to sucrose due to the bitter aftertaste of steviol glycosides [28] and high molecular weight.

The aim of this study was to investigate the effect of pretreatment performed by a combined method based on blanching, ultrasound, and vacuum application on the kinetics of osmotic dehydration in two solutions (61.5% sucrose solution and 30% sucrose solution with the addition of 0.1% of steviol glycosides). Moreover, selected quality properties of osmodehydrated cranberries as water activity, color and polyphenols, flavonoids, and anthocyanins content were evaluated.

## 2. Materials and Methods

### 2.1. Material

Swamp cranberry (Vaccinium oxycoccus) bought at the local market (Bronisze, Poland) was used as targeted matrix. Due to seasonality of the matrix, and to ensure reproducible results, cranberries from one batch, harvested in October 2017, have been frozen in a blast cabinet Irinox Shock Freezer HCM 51.20 (Irinox, Treviso, Italy) at a temperature of −25 °C for 5 h. Frozen fruits were stored at −18 °C no longer than 3 months. Only intact fruits of dark red coloring were used in the research.

### 2.2. Processing

#### 2.2.1. Blanching

Cranberries are a difficult material to process due to their hard peel. In order to ensure proper mass transfer during OD, the material was blanched until the peel broke. Blanching was performed at a temperature of 90 °C for 5 min [29]. The frozen material, weighed on a laboratory scale (RADWAG, Radom, Poland) with an accuracy of ± 0.01 g, and a distilled water were prepared in a ratio of 2:1 (w/w). After bringing the water to boiling, frozen cranberry fruits were placed in the water, which allowed them to reach the process temperature. During blanching, temperature was controlled by measuring the temperature. After 5 min of blanching, cranberry fruits were drained, dried on a filter paper, and re-weighed.

#### 2.2.2. Combined Pretreatment

Prior to the unconventional processing, cranberry fruits were weighed on a laboratory scale (RADWAG, Radom, Poland) with an accuracy of ±0.01 g and placed in beakers. Blanched cranberries were flooded with osmotic solution in 1:4 ratio. Afterwards, fruits followed unconventional pretreatment with the use of: Sonication—ultrasounds at 21 kHz frequency and 180 W power (ultrasound intensity equal to 3.6 W/g) generated by ultrasonic bath (MKD-3 ULTRASONIC) were applied [30]. Time of sample treatment amounted to 20 min [5,14], and during the ultrasound application changes in temperature of surrounding solution were below 1 °C. Reduced pressure—a specifically prepared desiccator was used in pretreatment to lower the pressure by 300 mmHg (~40 kPa) by vacuum pump. After reducing the pressure (lowering the pressure down to abovementioned value lasted for 20 s), the pretreatment took 10 min and then the vacuum was cut, which took around 5 s.

The unconventional treatment was carried out in duplicate and applied in three variants:

1. A blanched material was placed in osmotic solution and the pressure was lowered three times in 10 min intervals (BL_3 × 10 v),
2. A blanched material was placed in osmotic solution and subjected to lowered pressure for 10 min, then sonicated for 20 min (BL_10 v 20 us),
3. A blanched material was placed in osmotic solution and sonicated for 20 min, afterwards it was subjected to lowered pressure for 10 min (BL_20 us 10 v).

All analyzed samples were summarized in Table 1 with their related abbreviations.

#### 2.2.3. Osmotic Dehydration (OD)

OD was conducted in two solutions: 61.5% sucrose solution and 30% sucrose solution with addition of 0.1% steviol glycosides. A sucrose solution of 61.5% concentration was chosen since it is usually used as a standard solution [2,5,31]. However, a solution with the addition of steviol glycosides was used to reduce sugar content in dehydrated fruits. Due to the fact that steviol glycosides are up to 300 times sweeter than sucrose, the addition of little amount (0.1%) of this sweetener is enough to maintain its sweetness comparable with fruits dehydrated in a standard solution [25,32].

The pretreated fruits were placed in beakers and poured with osmotic solution in 1:4 ratio. OD was applied at a temperature of 40 °C for 72 h in a water bath with a stirrer at a rotation rate of 100 rotations per minute and at amplitude of 4. During OD, the process kinetics were determined after 1, 3, 6, 24, 48, and 72 h, measuring the mass changes, dry matter content, and water soluble solids. OD was performed in duplicate.

The analysis of cranberry fruits’ osmotic dehydration kinetics were determined based on water loss WL (kg H_2_O/kg of cranberry) and dry matter mass gain SG (kg d.m./kg of cranberry) [5]:

(1) $$\mathrm{WL}=\left(m_{o}X_{o}^{w}-m_{t}X_{o}^{w}\right)/m_{o}$$

(2) $$\mathrm{SG}=\left(m_{t}X_{t}^{ST}-m_{o}X_{o}^{ST}\right)/m_{o}$$

where m_o_—initial mass of cranberry prior to dehydration [kg], m_t_—final mass of cranberry after dehydration [kg], X_o_^w^, X_o_^ST^—water and dry matter content prior to dehydration [kg/kg], X_t_^w^, X_t_^ST^—water and dry matter content after dehydration [kg/kg].

### 2.3. Cranberries Analysis

#### 2.3.1. Dry Matter Content

Dry matter content was determined by weighing differently pretreated cranberries at 70 °C for 20 h [33]. The analysis was performed in triplicate.

#### 2.3.2. Water Soluble Solids (Brix Index)

Water soluble solids were determined by squeezing the juice out of fruits with linen material. Squeezed extract was placed in refractometer glass ATAGO PAL-3, and results were in Brix degrees (°Bx) [2]. The measurement was done in a triplicate.

#### 2.3.3. Water Activity

Water activity of cranberries were determined after a given time of OD. The measures were held with hygrometer Aqua Lab CX-2 (Decagon Devices Inc., United States) at 25 °C in three repetitions for a few randomly selected fruits [34].

#### 2.3.4. Color

The color test of pretreated and dehydrated samples were performed with the use of chromameter of CM-5 type by Konica Minolta (Japan), with a reflection method in CIE L*a*b* system of the following parameters: Light source D65, angle 8 °C, standard observer CIE 2°. In CIE system L*, parameter refers to sample lightness, while a* coordinate denotes share of green (-) and red color (+), and b* coordinate describes share of blue (-) and yellow color (+). Prior to measurement the apparatus was black and white calibrated [35]. The measurement was repeated five times for each sample.

The absolute color difference was calculated based on the following equation:

(3) $$\Delta E=\sqrt{{\left({\Delta L}^{*}\right)}^{2}+{\left({\Delta a}^{*}\right)}^{2}+{\left({\Delta b}^{*}\right)}^{2}}$$

where ΔL*, Δa*, Δb* — indicator of color difference in comparison to blanched cranberry fruits.

#### 2.3.5. Bioactive Compounds

Bioactive compounds as polyphenols, flavonoids, and anthocyanins content were measured using spectrophotometric methods according to Nowacka et al. [30]. The analyses were performed in triplicate. Polyphenolic content was expressed in mg of gallic acid in 100 g dry matter, flavonoid content in mg of quercetin in 1 g of dry matter, and anthocyanin content in mg of cyanidin-3-glucoside per 100 g of fresh mass.

### 2.4. Statistical Analysis

One-way analysis of variance (ANOVA) was performed in order to determine the impact of pretreatment and osmotic solution on the physical and chemical properties of blanched cranberry fruits. A detailed comparison allowed for a split of samples into homogenic groups. For this purpose, Duncan test was used with confidence interval of 95% (significance level α = 0.05). Moreover, all investigated variables were used to perform cluster analysis using Ward method as a criterion for agglomeration and expressing the results using Euclidean distance. The analyses were carried out in Statistica 2013 software.

## 3. Results and Discussion

### 3.1. Kinetics of Osmotic Dehydration

OD causes two different types of mass changes. The first one is linked with water removal from dehydrated material towards solution with a higher osmotic potential. The second phenomenon occurring simultaneously is impregnation of the osmotic substance into the processed material [36,37]. Along with the progress of the OD, the mass transfer is hindered, due to the equalization of chemical potentials between material and solution. The intensity of this phenomenon is described by OD kinetics, by the means of water loss (WL) and soluble solid gain (SG) in osmodehydrated material during the process [5].

**Figure 1:**
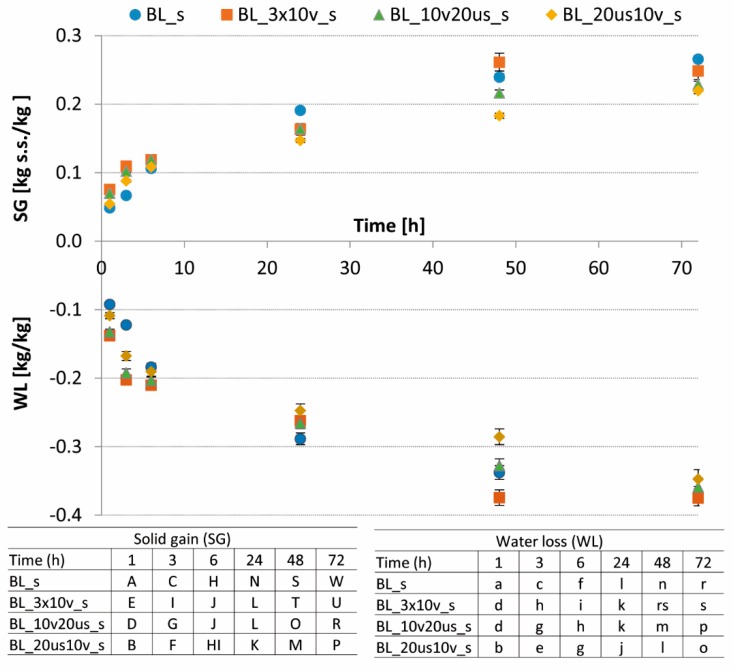
Water loss (WL) and solid gain (SG) as a function of the osmotic dehydration time of different treated cranberries in 61.5% sucrose solution. Different letter means significant difference by the Duncan test (*p* < 0.05).

**Figure 2:**
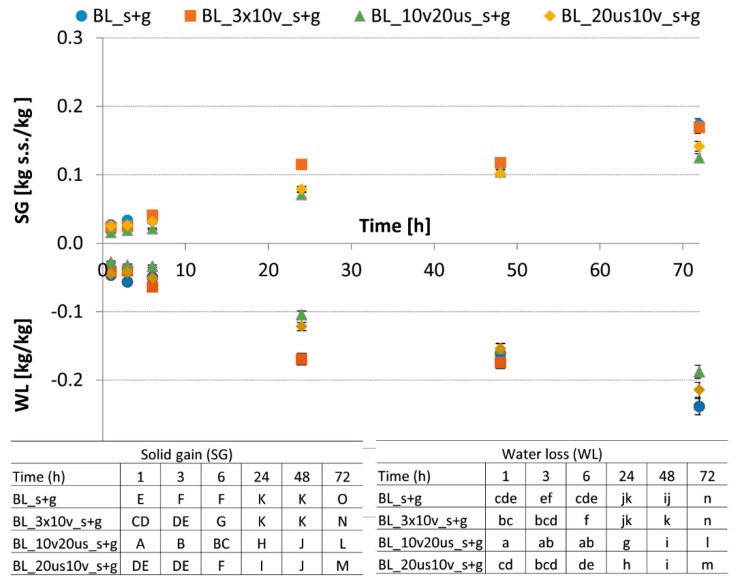
Water loss (WL) and solid gain (SG) as a function of the osmotic dehydration time of different treated cranberries in 30% sucrose solution with 0.1% addition of steviol glycosides. Different letter means significant difference by the Duncan test (*p* < 0.05).

During OD, an increase of WL was observed for both osmotic solutions—sucrose and sucrose with the addition of steviol glycosides (Figure 1 and Figure 2). In 61.5% of sucrose solution during the whole dehydration process a decrease of the water content in cranberries was observed (Figure 1). The WL was in the range of 0.35 kg H2O/kg (when sonication and reduced pressure treatment were used—BL_20 us 10 v_s) to 0.38 kg H2O/kg (when vacuum treatment was repeated three times—BL_3 × 10 v_s). The utilization of combined pretreatment significantly influenced the course of OD. The highest WL after 72 h of OD was noted after combined pretreatment with blanching and triplicate lowering of the pressure (BL_3 × 10 v_s). In turn, Nowacka et al. [32] reported that US applied as a pretreatment before OD increases the WL during OD, irrespective of the osmotic solution.

The use of 30% of sucrose with the addition of steviol glycosides caused a loss of water, which after 72 h of the process ranged from 0.19 to 0.25 kg H2O/kg, depending on the combined treatment used (Figure 2). The highest WL in the final stage of the OD was noted in the case of cranberry fruits subjected to triple vacuum treatment before the OD process (BL_3 × 10 v_s + g) and to only blanching (BL_s + g). However, in the case of combined methods in which US was used (BL_10 v 20 us_s + g; BL_20 us 10 v_s + g) a statistically lower water loss after 72 h of the OD was noted. Also, Sulistyawati et al. [38] noted statistically unchanged WL after reduced pressure treatment, compared with classical OD. A reverse dependence was noted by Feng et al. [39] for garlic but for a different osmotic solution—25% NaCl. The authors reported a significant increase in water loss as a result of both single vacuum (VOD) and ultrasound pretreatments (UOD), as well as for combined method—vacuum treatment with ultrasonic-assisted osmotic dehydration (VUOD) of garlic. As they have shown, the impact of vacuum treatment was more noticeable than the effect of sonication. However, when the combined method VUOD was used, the highest water loss was noted. Koubaa et al. [13] stated that US pretreatment accelerate the mass and heat transfer, which is linked with a cavitation effect provoked by sonication. Goula et al. [40] also observed a higher WL after US treatment before OD and US-assisted OD of potatoes in NaCl solution. Their findings and the results obtained herein suggest that the impact of the treatment was connected with the utilized osmotic solution.

With the progressing of the OD process, the content of dry matter increases, which is related to the penetration of substances from osmotic solution to material [36]. A solid gain (SG) after 72 h of the process performed in 61.5% sucrose was in the range of 0.22 kg d.m./kg of cranberry for BL_20 us 10 v_s to 0.27 kg d.m./kg of cranberry for BL_s (Figure 1). In turn, in the case of the osmotic solution with a lower sugar content (s + g) a lower solid gain was noted (Figure 2), which was connected with a lower driving force of the OD [38]. Similarly, as in the case of 61.5% sucrose, when s + g solution was used (Figure 2), the highest SG was observed for sample blanched before OD (BL_s + g), which reached the value of 0.17 kg d.m./kg of cranberry. Simultaneously, the lowest solid gain after OD, amounted to 0.13 kg d.m./kg of cranberry, and was characterized for material treated with US followed by reduced pressure treatment (BL_20 us 10 v_s + g). Statistical analysis revealed significant changes in SG as a result of both the time of OD and the type of different pretreatments. Contrary, Feng et al. [39] noted an irrelevant increase of SG in garlic dehydrated in NaCl after vacuum and the majority of US treatments [32]. Similar results were presented also in our study for cranberry pretreated by US and dehydrated in different osmotic solutions. Allahdad et al. [41] concluded that the impact of US-assisted OD of pomegranate arils on the SG was dependent on the applied US frequency. When the 40 kHz was used the SG was significantly higher than in the case of both 21 kHz and material without US treatment. This suggests that the treatment type and the treatment parameters should be adjusted for each material individually.

### 3.2. Physical Properties—Dry Matter Content, Water Activity, Water Soluble Compounds, and Color

Depending on the variety, fresh cranberry fruits are characterized by a dry substance ranging from 12.1% to 14.5%, and water-soluble compounds are from 7 to 9.4 °Bx [42]. In the case of blanched fruits, similar results of water-soluble compounds (9.3 ± 0.2 °Bx) and dry matter content (14.6 ± 0.1%) were observed (Table 2). However, the combined pretreatment, consisting of the application of reduced pressure or combining reduced pressure with ultrasound conducted in an osmotic solution, caused a significant increase in the dry matter and Brix index in all cases. The increase in these parameters was related to the penetration of the components in the osmotic solution used during the pretreatment [36]. Pretreatment in a sucrose solution resulted in an increase in the dry matter content by 32.2% to 39.7%, depending on the combination used, whereas in the solution with the addition of steviol glycosides by 10.3% to 13.7%, compared to blanched cranberries. For water soluble compounds, a greater increase of brix index in cranberry fruits was observed while a higher concentration of sucrose solution was used. Blanched fruits treated with ultrasound before or after the application of reduced pressure treatment in sucrose solution (BL_20 us 10 v and BL_10 v 20 us_s) were characterized by the highest water-soluble compounds of 15.9 ± 0.4 and 15.9 ± 0.5° Bx, respectively. The higher increase in the brix index in the ultrasound-treated tissue could be related to the intensification of the OD process enhanced with ultrasound waves [42] and with the changes of the plant tissue subjected to sonication [43]. However, such changes were not noticed during the OD process in the solution with a lower sucrose content with the addition of steviol glycosides.

After 72 h of OD process a significant increase in the dry matter content and Brix index was observed, in the range from 28.9% to 45.9% and from 22.4 to 43.1 °Bx, respectively. The highest content of these parameters after the OD process was found in the blanched material (BL_s_72 h), and then in the tissue subjected to reduced pressure treatment (BL_3 × 10 v_s_72 h). In the case of combined pretreatment, the dry matter content was slightly lower and it was 45.2% for BL_10 v 20 us_s_72 h and 41.9% for BL_20 u s10 v_s_72 h. The similar effect, with lower values, was observed for cranberries subjected to the OD process in sucrose solution with the addition of steviol glycosides, which was linked to the lower driving force of the process [36].

Water activity is one of the key parameters determining the durability of food, affecting the survival and growth of microorganisms, enzymatic reactions, and oxidation. Water activity for most food products of plant origin is close to 1 [44]. For blanched and differently treated cranberries, water activity was in the range from 0.934 to 0.966 (Table 1). After the OD process a significant decrease in water activity for both solutions was noticed. In a solution with a higher concentration of sugar (61.5%) the water activity was in the range of 0.862 (BL_3 × 10 v_s_72 h) to 0.896 (BL_20 us 10 v_s_72 h). OD allowed reducing the water activity which limits only the growth of bacteria [44]. However, in the case of OD in a 30% sucrose solution with the addition of steviol glycosides, a smaller decrease was observed and the water activity reached a value from 0.914 (BL_3 × 10 v_s + g_72 h) to 0.919 (BL_20 us 10 v_s + g_72 h).

Color is one of the most important features of the product, which consumers pay special attention to [35]. Cranberry is characterized by a red color, which changes during processing [33]. The blanched fruits were characterized by L*, a*, and b* parameters equal to 9.1, 51.8, and 15.7, respectively. The color changes were not unambiguous (Table 1). The use of reduced pressure treatment and combined treatment with US, and lowered pressure resulted in a significant increase in lightness (L*) and a decrease in the share of red color (a*), compared to the only blanched sample. OD processing caused further lightening and lower value of a* parameter, which is related to mass transfer form tissue to the surrounding solution. What is worth mentioning is that higher changes were observed for combined treatment, which could be linked to the acceleration of OD processes by the application of reduced pressure treatment and US [13,35]. Moreover, a significant increase in the total color difference ΔE was found with the value higher than five, which confirms that the treatment performed had a significant effect on the overall change in the color of the product. However, higher changes were noted for the samples processed in 61.5% sucrose solution, probably due to the higher driving force of OD process. The changes of color of OD fruits pretreated by blanching and blanching combined with reduced pressure in comparison to intact, blanched material are also related to higher sugar concentration. The presence of sugar affects the light reflection and thus changes the lightness, redness, and the yellowness of the material. Moreover, as described and discussed further, dehydrated samples contained less of the pigment compounds such as anthocyanins or flavonoids, which explains the smaller values of a* parameter when compared to the reference (BL) material. The sequence of application of reduced pressure treatment and ultrasound did not play a significant role in shaping the color of fruits after OD.

### 3.3. Bioactive Compounds

**Figure 3:**
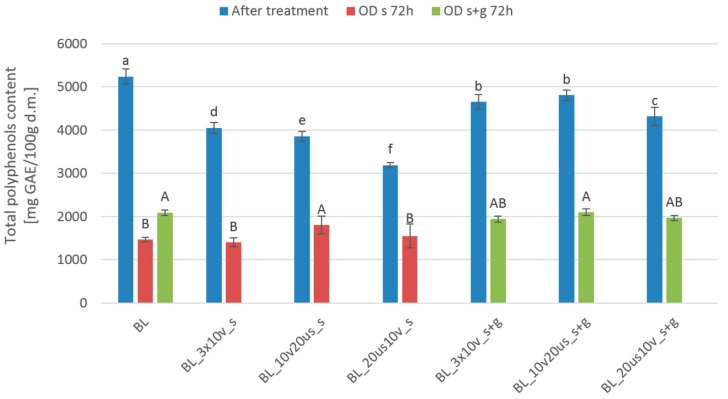
Total phenolic content in different treated cranberries: Blanched (BL), reduced pressure (BL_3 × 10 v), reduced pressure with sonication treatment (BL_10 v 20 us), sonication treatment with reduced pressure (BL_20 us 10 v), and after osmotic dehydration (OD) in 61.5% sucrose solution (s) and in 30% sucrose solution with 0.1% addition of steviol glycosides (s + g). Different lowercase letters means significant difference by the Duncan test (*p* < 0.05) for samples after treatment, and different capital letter means significant difference by the Duncan test (*p* < 0.05) for samples after 72 h osmotic dehydration.

Cranberries contain a lot of different bioactive compounds [21]. Figure 3 presents the total phenolic content of cranberry fruits subjected to different pretreatment before and after OD. The highest total phenolic content (5241 ± 179 mg/100g d.m.) was stated for blanched samples. Combination of blanching with other pretreatment methods like reduced pressure treatment or sonication led to a decrease of phenolic compounds in comparison to the blanched material. In general, processing performed with 61.5% sucrose solution lead to smaller phenolic content of cranberries in comparison to fruits processed in a ternary solution composed of sucrose and steviol glycosides. Such situations are associated with higher leakage of polar phenolics to the surroundings during treatment in a more concentrated solution. The difference in phenolic content between samples processed with concentrated sucrose and ternary solution varied from 22.7% to 39.2% for material coded as BL_3 × 10 v_s and BL_20 us 10 v_s, respectively. The introduction of additional processing steps like reduced pressure treatment and sonication contributed to a significant reduction of phenolics in investigated fruits in comparison to less complicated operations. One of the possible explanations of such behavior is related to a sonoporation phenomenon which increases cell membrane permeability [45]. On the one hand, the sonoporation leads to degradation of the cellular structure and thus it may enhance the extractability of bioactive compounds. On the other hand, it implies improved mass transfer between fruits and osmotic medium which can positively impact on water loss but negatively influence to the phenolic phase promoting its leaching. Another explanation of smaller phenolic content of samples subjected to treatment with sonication is associated with the free radical formation during US application which can lead to degradation of antioxidants [46,47]. The impact of ultrasound on enzymatic activity is ambiguous and the effect of US on enzymes seems to depend on the matrix, treatment parameters, and type of the enzyme alike [48,49]. For instance, it has been reported that the combination of ascorbic acid with ultrasound was successful in the inactivation of polyphenoloxidase and peroxidase, whereas individual application of ascorbic acid and sonication did not give the desired inactivation of abovementioned enzymes in apples [50]. In turn, ultrasound treatment of diluted avocado puree increased activity of polyphenols oxidase by 180% depending on treatment [51]. Based on the results presented in the current study it can be stated that ruptured cellular structure and liberated chemicals were more susceptible for enzymatic degradation as the activity of enzymes was modified by sonication. This explanation is also supported by the fact that the phenolics content of fruits subjected only to blanching and reduced pressure treatment before OD was lower when compared to samples treated by a combination of blanching, vacuum treatment, and sonication. It is, furthermore, reported that ultrasounds can provoke conformation changes of enzymes, lead to dissociation of enzyme aggregates, promote collisions between substrate-enzyme, or activate latent enzymes of the tissue [49]. However, when ultrasound was used as treatment for 30 min for blanched cranberries it resulted in higher total phenolic content [52] than when combined methods with reduced pressure were used. OD for 72 h led to a further decrease of phenolics. Combined treatment in most cases did not cause significant differences in comparison to blanched osmodehydrated samples. Only fruits subjected to reduced pressure combined with sonication and dehydrated in 61.5% sucrose solution exhibited significantly higher phenolic content (1802 mg GAE/100 g d.m.) in comparison to the blanched material processed in the same osmotic medium (1465 mg GAE/100 g d.m.).

**Figure 4:**
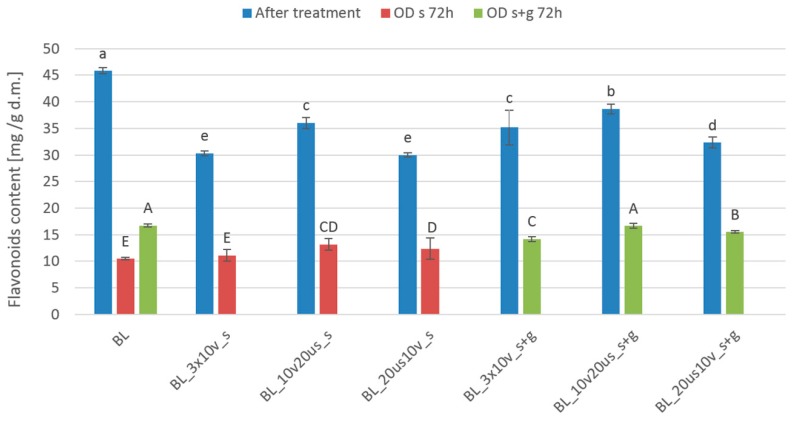
Flavonoids content in different treated cranberries: Blanched (BL), reduced pressure (BL_3 × 10 v), reduced pressure with sonication treatment (BL_10 v 20 us), sonication treatment with reduced pressure (BL_20 us 10 v), and after osmotic dehydration (OD) in 61.5% sucrose solution (s) and in 30% sucrose solution with 0.1% addition of steviol glycosides (s + g). Different lowercase letters means significant difference by the Duncan test (*p* < 0.05) for samples after treatment, and different capital letters means significant difference by the Duncan test (*p* < 0.05) for samples after 72 h osmotic dehydration.

Flavonoids content of investigated cranberries ranged from 30.0 to 45.8 mg/g d.m. and from 10.5 to 16.7 mg/g d.m. before and after 72 h of OD, respectively (Figure 4). The highest flavonoids content was stated for blanched samples before OD. The use of combined treatment decreased flavonoids content from 16% to 35% in the case of samples treated by sonication combined with reduced pressure in 61.5% sucrose solution (BL_20 us_10 v_s) and by reduced pressure treatment combined with sonication in 30% sucrose solution with addition of steviol glycosides (BL_10 v_20_us_s + g). As in the case of phenolic compounds, OD for 72 h significantly decreased flavonoids content regardless of the treatment protocol. In general, samples osmodehydrated in ternary solution (s + g) were characterized by higher flavonoids content than the samples processed in 61.5% sucrose solution (s). For instance, flavonoids content of fruits treated by reduced pressure followed by sonication in 30% sucrose solution with the addition of steviol glycosides was 26.2% higher in comparison to the material processed by the same method but in 61.5% sucrose solution. Such difference was most probably linked to the worse mass transfer of less concentrated solution [53]. It is worth emphasizing that samples subjected to sonication exhibited a higher concentration of flavonoids in comparison to the fruits treated only by reduced pressure treatment. What is more, when sonication was preceded by reduced pressure application, the retention of flavonoids was higher. Similar results were obtained for blanched cranberries treated with ultrasound for 30 min in sucrose solution [52]. These results point out that the effect of ultrasound on improvement of extractability is more pronounced when air in the extracellular space is replaced by liquid. This explanation fits to the data reported by Liu et al. [54] who found out that cavitation is enhanced in degassed water.

**Figure 5:**
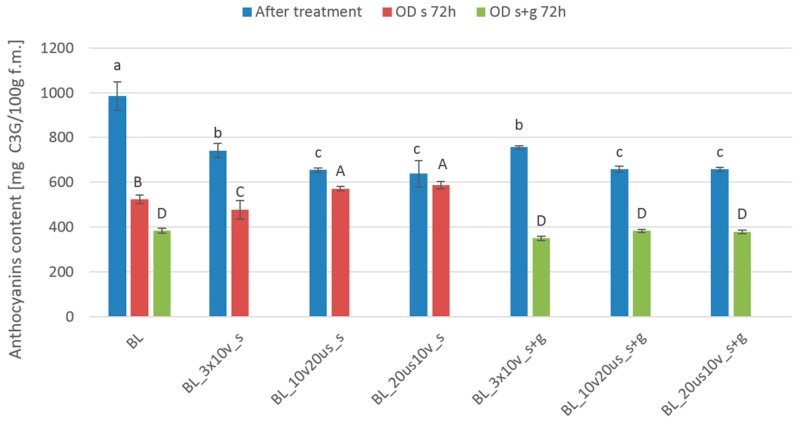
Anthocyanins content in different treated cranberries: Blanched (BL), reduced pressure (BL_3 × 10 v), reduced pressure with sonication treatment (BL_10 v 20 us), sonication treatment with reduced pressure (BL_20 us 10 v), and after osmotic dehydration (OD) in 61.5% sucrose solution (s) and in 30% sucrose solution with 0.1% addition of steviol glycosides (s + g). Different lowercase letters means significant difference by the Duncan test (*p* < 0.05) for samples after treatment, and different capital letters means significant difference by the Duncan test (*p* < 0.05) for samples after 72 h osmotic dehydration.

Figure 5 presents the anthocyanins content of fruits subjected to different treatments. Samples before OD were characterized by higher anthocyanins content, which ranged from 638 to 985 mg c3g/100 g f.m. The use of combined treatment decreased anthocyanins content in comparison to blanched material. Samples subjected to triple pressure reduction were characterized by significantly higher concentration of anthocyanins in comparison to samples treated by reduced pressure and sonication, regardless of the type of osmotic solution. However, longer sonication (30 min) of blanched cranberries resulted in higher content of anthocyanins, as mentioned in our previous study [52]. What is interesting is that after OD the trend was reversed, which indirectly confirms the ambiguous character of sonication either to improve extractability of bioactive compounds or to cause their degradation. The utilization of 61.5% sucrose solution resulted in better anthocyanins preservation than the use of low concentrated (30%) sucrose solution with the addition of steviol glycosides. These findings fall into place with a protective role of sugar on anthocyanins stability due to the decreased water mobility as demonstrated by Tsai et al. [55] and Watanabe et al. [56].

### 3.4. Cluster Analysis

**Figure 6:**
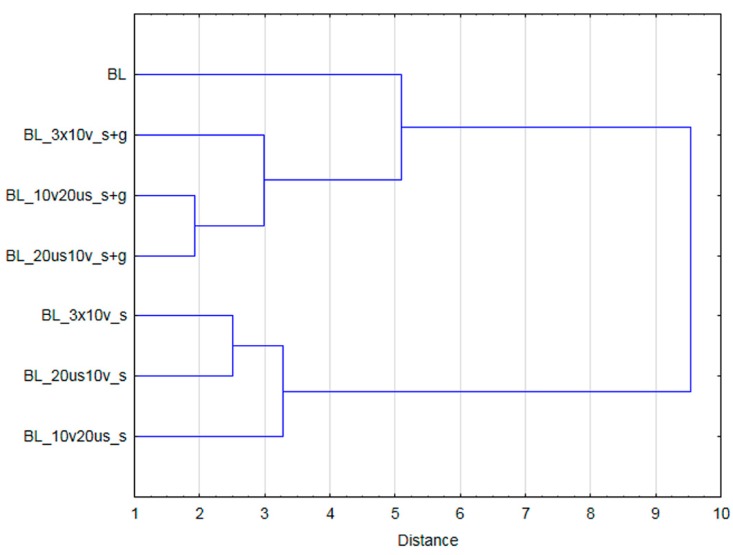
The results of cluster analysis (considering all analyzed variables) for samples not subjected to osmotic dehydration (BL - blanched material).

**Figure 7:**
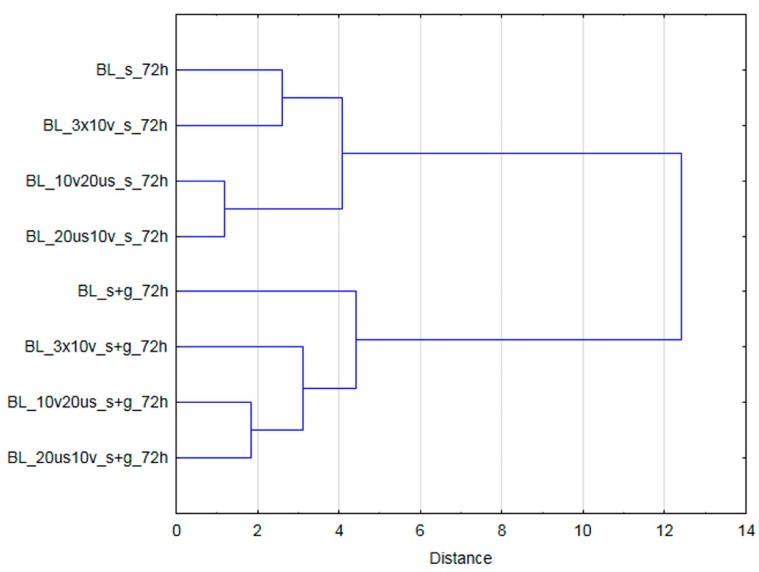
The results of cluster analysis (considering all analyzed variables) for samples subjected to osmotic dehydration.

Figure 6 and Figure 7 present the results of cluster analysis performed on the basis of all investigated variables for samples not subjected and subjected to OD, respectively. Samples before OD were divided into three groups. The first cluster was composed of only blanched material, whereas second and third group was built by samples subjected to treatment performed in ternary (30% of sucrose with the addition of steviol glycosides) and 61.5% sucrose solution, respectively. Based on the cluster analysis results it can be stated that samples subjected to dehydration in less concentrated solutions were more similar to blanched material than samples processed in a concentrated sucrose solution. For materials after dehydration, clusters were formed upon the type of osmotic medium as well. It means that all fruits dehydrated in 61.5% sucrose solution formed one group while processing in 30% sucrose solution with the addition of steviol glycosides resulted in formation of a separate group.

## 4. Conclusions

Unconventional pretreatment of cranberries caused a significant increase of osmotic dehydration effectiveness, particularly in the case of a material dehydrated in 61.5% sucrose solution, previously blanched, submitted to 20 min sonication, followed by low pressure application.

Cranberries subjected to combined treatment, in particular to ultrasounds, had comparable or higher polyphenolic, anthocyanin and flavonoids content than a blanched tissue subjected to osmotic dehydration. Taking into account evaluated physical and chemical properties of dehydrated cranberries and the osmotic dehydration process it has been concluded that the best combined pretreatment method was a 20 min sonication followed by a 10 min lowered pressure treatment. However, further optimization studies are required.

## Figures and Tables

**Table 1:** Abbreviations of all researched samples of cranberries.

Treatment	Abbreviations	
**After treatment, before osmotic dehydration**		
Blanched material	BL	
	Osmotic solution	
	Sucrose 61.5% (s)	Sucrose 30% with 0.1% steviol glycosides addition (s + g)
Blanched material in osmotic solution under the pressure lowered three times in 10 min intervals (BL_3 × 10v)	BL_3 × 10 v_s	BL_3 × 10 v_s + g
Blanched material in osmotic solution subjected to lowered pressure for 10 min and sonicated for 20 min (BL_10 v 20us)	BL_10 v 20 us_s	BL_10 v 20 us_s + g
Blanched material in osmotic solution subjected to sonication for 20 min and to lowered pressure for 10 min (BL_20 us 10 v)	BL_20 us 10 v_s	BL_20 us 10 v_s + g
**After osmotic dehydration**		
Blanched material subjected to osmotic dehydration for 72 h (BL_72 h)	BL_s_72 h	BL_s + g_72 h
Blanched material in osmotic solution under the pressure lowered three times in 10 min intervals, and then subjected to osmotic dehydration for 72 h (BL_3 × 10 v_72 h)	BL_3 × 10 v_s_72 h	BL_3 × 10 v_s + g_72 h
Blanched material in osmotic solution subjected to lowered pressure for 10 min and sonicated for 20 min, and then osmotic dehydration for 72 h (BL_10 v 20 us_72 h)	BL_10 v 20 us_s_72 h	BL_10 v 20 us_s + g_72 h
Blanched material in osmotic solution subjected to sonication for 20 min and to lowered pressure for 10 min, and then osmotic dehydration for 72 h (BL_20 us 10 v_72 h)	BL_20 us 10 v_s_72 h	BL_20 us 10 v_s + g_72 h

BL - blanched material.

**Table 2:** Dry matter content, water activity, water soluble compounds, and color parameters in different treated cranberries, different letters in columns means significant difference by the Duncan test (*p* < 0.05).

Material	Dry Matter Content [%]	Water Activity [−]	Water Soluble Compounds [°Bx]	Color Parameters			
				L *	a *	b *	Total Color Difference ΔE
**BL**	**14.6 ± 0.1a**	**0.952 ± 0.009 ^fg^**	9.3 ± 0.2 ^a^	9.1 ± 0.7 ^a^	51.8 ± 0.6 ^i^	15.7 ± 0.7 ^bc^	−
**After treatment**							
**s solution**							
BL_3 × 10 v_s	19.3 ± 0.1 ^c^	0.937 ± 0.006 ^de^	14.5 ± 0.2 ^c^	16.1 ± 0.8 ^h^	40.7 ± 0.7 ^f^	17.2 ± 0.6 ^efg^	13.2 ± 0.8 ^c^
BL_10 v 20 us_s	20.4 ± 0.1 ^c^	0.950 ± 0.004 ^f^	15.9 ± 0.5 ^d^	21.1 ± 0.7 ^i^	35.0 ± 0.6 ^cd^	13.8 ± 0.7 ^a^	20.8 ± 0.5 ^h^
BL_20 us 10 v_s	19.3 ± 0.1 ^c^	0.934 ± 0.005 ^e^	15.9 ± 0.4 ^d^	13.8 ± 1.3 ^d^	35.5 ± 0.6 ^d^	14.5 ± 0.8 ^a^	17.1 ± 0.7 ^ef^
**s + g solution**							
BL_3 × 10 v_s + g	16.6 ± 0.1 ^b^	0.952 ± 0.007 ^fg^	11.8 ± 0.2 ^b^	14.7 ± 0.4 ^e^	43.0 ± 0.9 ^g^	14.1 ± 0.4 ^a^	10.7 ± 0.6 ^b^
BL_10 v 20 us_s + g	16.3 ± 0.3 ^b^	0.963 ± 0.003 ^g^	11.9 ± 0.1 ^b^	12.9 ± 0.8 ^c^	46.5 ± 0.8 ^h^	16.4 ± 0.7 ^bcd^	6.6 ± 0.8 ^a^
BL_20 us 10 v_s + g	16.1 ± 0.1 ^b^	0.966 ± 0.002 ^fg^	11.9 ± 0.1 ^b^	11.0 ± 0.5 ^b^	47.3 ± 0.7 ^h^	18.6 ± 0.6 ^h^	5.8 ± 0.6 ^a^
**After osmotic dehydration**							
**s solution**							
BL_s_72 h	45.9 ± 0.6 ^h^	0.898 ± 0.018 ^b^	43.1 ± 0.4 ^g^	14.8 ± 0.7 ^e^	34.2 ± 0.9 ^bc^	16.7 ± 0.8 ^cde^	18.6 ± 0.8 ^g^
BL_3 × 10 v_s_72 h	45.2 ± 0.8 ^h^	0.862 ± 0.014 ^a^	42.5 ± 0.5 ^g^	16.2 ± 0.5 ^h^	34.0 ± 0.8 ^b^	17.7 ± 0.7 ^g^	19.3 ± 0.7 ^g^
BL_10 v 20 us_s_72 h	43.1 ± 0.7 ^g^	0.895 ± 0.002 ^b^	36.1 ± 0.8 ^h^	15.6 ± 0.7 ^fgh^	31.9 ± 1.1 ^a^	16.4 ± 0.5 ^bcd^	21.0 ± 1.0 ^h^
BL_20 u s10 v_s_72 h	41.9 ± 0.7 ^g^	0.896 ± 0.009 ^b^	36.1 ± 0.4 ^h^	15.4 ± 1.1 ^ef^	31.8 ± 0.8 ^a^	15.9 ± 1.0 ^bc^	21.0 ± 0.7 ^h^
**s + g solution**							
BL_s + g_72 h	34.2 ± 1.1 ^f^	0.916 ± 0.009 ^c^	22.4 ± 0.2 ^e^	12.9 ± 0.7 ^c^	37.5 ± 1.0 ^e^	15.6 ± 0.9 ^b^	14.9 ± 0.9 ^d^
BL_3 × 10 v_s + g_72 h	32.9 ± 0.9 ^f^	0.914 ± 0.004 ^c^	26.5 ± 0.8 ^g^	16.1 ± 0.1 ^gh^	37.3 ± 2.9 ^e^	15.6 ± 2.3 ^b^	16.3 ± 2.6 ^e^
BL_10 v 20 us_s + g_72 h	28.9 ± 1.0 ^d^	0.928 ± 0.005 ^d^	23.0 ± 1.5 ^e^	15.9 ± 0.3 ^fgh^	35.7 ± 0.5 ^d^	17.5 ± 0.3 ^fg^	17.6 ± 0.8 ^f^
BL_20 us 10 v_s + g_72 h	31.0 ± 0.9 ^e^	0.919 ± 0.005 ^c^	25.6 ± 0.9 ^f^	15.4 ± 0.2 ^efg^	34.0 ± 0.9 ^b^	16.6 ± 1.6 ^cde^	19.0 ± 0.5 ^g^
